# Particle Focusing in a Straight Microchannel with Non-Rectangular Cross-Section

**DOI:** 10.3390/mi13020151

**Published:** 2022-01-20

**Authors:** Uihwan Kim, Joo-Yong Kwon, Taehoon Kim, Younghak Cho

**Affiliations:** 1 Department of Mechanical Design and Robot Engineering, Seoul National University of Science & Technology, 232 Gongneung-ro, Nowon-gu, Seoul 01811, Korea; them10@naver.com (U.K.); wock2003@naver.com (J.-Y.K.); 2 Department of Mechanical System Design Engineering, Seoul National University of Science & Technology, 232 Gongneung-ro, Nowon-gu, Seoul 01811, Korea

**Keywords:** non-rectangular microchannel, particle focusing, Newtonian fluid, viscoelastic fluid

## Abstract

Recently, studies on particle behavior under Newtonian and non-Newtonian fluids in microchannel have attracted considerable attention because particles and cells of interest can be manipulated and separated from biological samples without any external force. In this paper, two kinds of microchannels with non-rectangular cross-section were fabricated using basic MEMS processes (photolithography, reactive ion etching and anisotropy wet etching), plasma bonding and self-alignment between two PDMS structures. They were used to achieve the experiments for inertial and elasto-inertial particle focusing under Newtonian and non-Newtonian fluids. The particle behavior was compared and investigated for different flow rates and particle size in the microchannel with rhombic and equilateral hexagonal cross section. We also investigated the influence of Newtonian fluid and viscoelastic fluid on particle migration in both microchannels through the numerical simulation. The experimental results showed the multi-line particle focusing in Newtonian fluid over a wide range of flow rates, but the single-line particle focusing was formed in the centerline under non-Newtonian fluid. The tighter particle focusing appeared under non-Newtonian fluid in the microchannel with equilateral hexagonal cross-section than in the microchannel with rhombic cross section because of the effect of an obtuse angle. It revealed that particles suspended in the channel are likely to drift toward a channel center due to a negative net elasto-inertial force throughout the cross-sectional area. Simulation results support the present experimental observation that the viscoelastic fluid in the microchannel with rhombic and equilateral hexagonal cross-section significantly influences on the particle migration toward the channel center owing to coupled effect of inertia and elasticity.

## 1. Introduction

Recently, there have been numerous studies to separate particles and cells from complex and heterogeneous samples in medical, biological, and chemical fields in microfluidic channels as a passive particle manipulating technique. Although this technique does not require any external force, it allows a high performance over a wide range of flow rates, high resolution, and a better efficiency in processing time with reduced sample consumption in comparison with traditional separation methods such as flow cytometry [1,2,3,4,5].

One part of the inertial microfluidics field has explored for manipulating particles and its resulting separation in Newtonian fluid such as deionized (DI) water and PBS solution [6,7]. Di Carlo explained the focusing position of particles and cells according to the cross-section shape of the microfluidic channel under Newtonian fluid, which is determined by the balance of the two inertial lift forces: shear-gradient lift force and wall-effect lift force [4]. Several groups developed various inertial microfluidic devices for particle focusing, cell sorting, and blood sample preparation [2,8,9,10,11,12] utilizing specific microchannels such as serpentine, asymmetric reverse wavy, high aspect ratio and contraction–expansion microchannel. Li et al. developed a continuous and high-throughput microfluidic device for white blood cell separation utilizing the differential inertial focusing of particles in serpentine microchannels [2]. Ai et al. presented a novel geometric channel design, an asymmetric reverse wavy microchannel, for sheathless inertial particle focusing and cell sorting, which can minimize the use of fluid pumps [8]. Shen et al. developed a multistage microfluidic device for continuous label-free separation and enrichment of rare cells from blood samples using a unique combination of inertial microfluidics and steric hindrance [9]. Han et al. used a label-free, shear-modulated inertial microfluidic system with high aspect ratio microchannels coupled with pinched flow dynamics to isolate ring-stage malaria parasites from lysed blood containing white blood cells [10]. Xiang et al. explored the particle focusing mechanisms in a symmetrical serpentine microchannel [11] and in different contraction–expansion ratio channels [12] with the numerical simulation and experiment validation. They believed that these hydrodynamic control of micro-particles promises to be an effective and efficient manipulation method for particle focusing and separation. Some reviews for secondary flow in inertial microfluidics were also introduced, which has been widely employed for fluid and particle manipulation, such as mixing, trapping, focusing and separation [13,14]. They introduced and discussed the mechanism, physics and usage of secondary flow on inertial particle migration in inertial microfluidics.

Another part in inertial microfluidics has focused on non-Newtonian fluids for particle separation. In non-Newtonian fluid regime, elastic force appears and significantly impacts on particle migration in addition to fluid inertia, resulting in elasto-inertial focusing [15,16,17]. To be specific, such elastic effect causes an emergence of the second normal stress differences that induce secondary flow in non-circular channel [18] and alters migrating behavior of inertial particles in three-dimensional space [19]. Various numerical and experimental studies have been conducted to evaluate the second normal stress with respect to the secondary flow and its corresponding particle focusing in different viscoelastic fluids as well as channel geometry [20,21,22,23,24]. Seo et al. studied for the impact of flow rate and shear-thinning property of viscoelastic fluids on particle focusing in a square duct microchannel by leveraging holographic microscopic technique [20]. They observed that particle focusing occurs when a synergetic combination of the elasticity and the inertia was achieved. This observation has been consistently reported in both numerical and experimental works with different elastic effect [21], particles [22] and channel geometry [16,17].

Meanwhile, since the cross-sectional shape of the channel significantly influences the inertial lift forces under Newtonian fluid [6,7] as well as the second normal stress difference and the secondary flow in viscoelastic regime under non-Newtonian fluid [25], migrating pattern of inertial particles should be also affected. However, only limited studies have been reported for the influence of cross-sectional geometry of microchannel on inertial focusing and elasto-inertial focusing due to challenges in fabrication of the microchannel. That is, despite decades of research on Newtonian and non-Newtonian fluids for particle manipulation, most studies have continued to use rectangular microchannels with different aspect ratio. Owing to the recent improvements in the microfabrication techniques including MEMS [6], mechanical micro-milling [7,26,27] and 3D printing [23,28], which could be used for fabricating micro-molds with various cross-sectional geometry, microchannels with non-rectangular cross-section (rectangle, triangle, semi-circle, circle, trapezoid and etc.) could be fabricated in combination with the conventional polydimethylsiloxane (PDMS) cast molding method. Kim et al. fabricated semi-circular and triangular microchannels using MEMS processes, which were used to demonstrate inertial focusing in a Newtonian fluid [6]. Low aspect-ratio triangular microchannel [7] and trapezoidal microchannel [26] were also fabricated to study the inertial focusing of microparticles and manipulate them. Raoufi et al. fabricated microchannels with various cross-sectional shapes using micro-milling and micro-molding, and studied the behavior of particles in viscoelastic fluids through experiments and simulations [27]. Through this, it was shown that the obtuse corner part of the cross-sectional shape of the microchannel influenced on the particle focusing. Tang et al. proposed a novel 3D-printed mold-removal method to fabricate microchannels with semi-elliptical and triangular cross-sections in which the mechanisms of elasto-inertial focusing were explored [23]. A high-quality wax 3D printer was also used to fabricate microchannels of arbitrary cross-sections analyzed to investigate the effects of viscoelasticity and superposition on the lateral migration of the particles [28]. However, Mashhadian et al. presented a method for fast prediction of focusing pattern in a straight channel with arbitrary cross-section [29]. To the best of our knowledge, however, microchannels with non-rectangular cross-section such as rhombus and hexagon were never realized until our study was presented [30]. In a previous study, we proposed a novel yet simple fabrication method of microchannels with various cross-sectional shapes, such as parallelogram, rhombus, pentagon and hexagon. Basic MEMS (microelectromechanical systems) processes such as photolithography, RIE (reactive ion etching) and anisotropic KOH (potassium hydroxide) wet etching was used to fabricate them, followed by self-alignment between Si structure and PDMS (polydimethylsiloxane) mold. Also, inertial and elasto-inertial particle migration in the rhombic microchannel were examined under Newtonian and non-Newtonian fluids in terms of the flow rate and particle size [24].

In this paper, we fabricated two kinds of non-rectangular PDMS microchannel with a rhombic and an equilateral hexagonal cross-section and carried out the experiments for the inertial and elasto-inertial particle focusing, respectively. The particle migrations in Newtonian fluid and non-Newtonian fluid were compared with each other according to the flow rate and particle size, and their effect on particle focusing position and focusing width were also investigated. Moreover, we evaluated the particle behavior in the microchannel with various cross-section under Newtonian or non-Newtonian fluid flow through the numerical simulation.

## 2. Materials and Methods

### 2.1. Micorchannels with Rhombic and Equilateral Hexagonal Cross-Section

**Figure 1:**
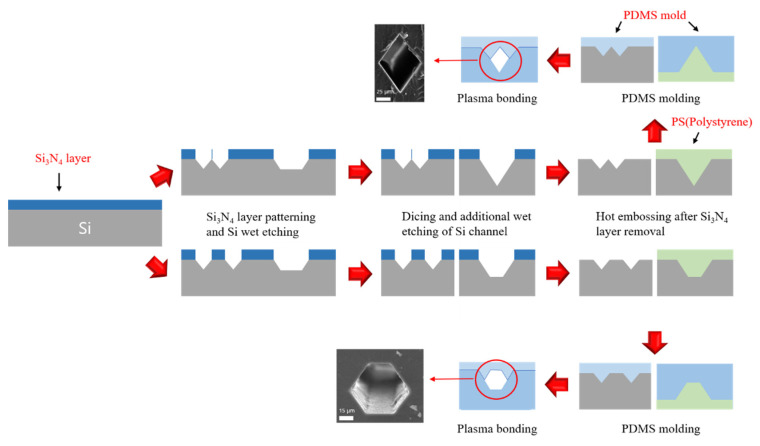
Schematic view of fabrication processes of the microchannel with rhombic and equilateral hexagonal cross-sectional shape and their cross-sectional SEM images [30].

The fabrication process of microchannels with rhombic and equilateral hexagonal cross-sectional shapes was already reported in the previous works [30]. Briefly explained, the Si masters for polystyrene and PDMS mold were formed using the basic MEMS processes such as photolithography, RIE and anisotropic KOH wet etching following the hot-embossing and conventional soft lithography technique. The PDMS microchannels from polystyrene mold and the PDMS mold were self-aligned and bonded by O_2_ plasma. As shown in Figure 1, equilateral hexagon has obtuse angles in all corners of the channel, but rhombus has two obtuse angles and two acute angles. In this paper, the particle behavior characteristics in a microchannel with equilateral hexagonal cross section will be evaluated by comparing it with a microchannel with rhombic cross section in which both obtuse and acute angles exist.

### 2.2. Sample Preparation and Experimental Setup

DI water (deionized water) and PEO (polyethylene oxide) solution was used as Newtonian solution and viscoelastic solution, respectively. To make 500 ppm PEO (PEO500) solution, PEO (M_w_ = ~2 MDa, Sigma-Aldrich, Burlington, MA, USA) was dissolved to DI water. 5 and 13 μm fluorescent polystyrene (PS) particles (Thermo Scientific inc., Waltham, MA, USA) were added to these experimental solutions (0.05~0.1 wt% concentration), respectively. Surfactant Tween 20 (Sigma-Aldrich, Burlington, MA, USA) was added into the suspensions at 0.1 wt% in order to prevent particle aggregation during the particle migration experiments. The estimated mean viscosity of PEO500 solution was about 1.85 mPa∙s (DI water: 0.89 mPa∙s), and its relaxation time was estimated from the previous empirical relaxation time (*λ*_e_) measured with a capillary breakup extensional rheometer (CaBER) [24]. The relaxation time for PEO500 solution is estimated to be 3.78 ms on the basis of the empirical formula.

Inverted optical microscope (BX-60, Olympus, Tokyo, Japan) was used to observe particle migration in the microchannel. For the flow control and image capture, a syringe pump (LEGATO 111, KD Scientific Inc., Holliston, MA, USA) and CMOS camera (Touptek Photonics Co., Ltd., Hangzhou, China) were used. The particles suspended in DI water and PEO solution were injected over the flow rates from 1 to 300 μL/min using syringe pump. To analyze the particle focusing behaviors, the bright-field images and fluorescent images were captured with the exposure times of 20 ms and 500 ms, respectively. All the analysis and post-processing on the captured images were performed using the open-source software ImageJ. In this paper, the optical intensity profile across the channel was extracted and fitted with a Gaussian distribution.

### 2.3. Simulation for Viscoelastic Flow

The main objective of the current simulation work is to explore the impact of the cross-sectional shape of the microchannel on inertial particle migration in viscoelastic regime and thus provide an underlying mechanism in particle migration as compared to the experimental counterpart in this study. Numerical simulation was carried out by utilizing finite-volume-based flow solver (ANSYS Fluent). To be specific, the equilibrium position of inertial particles is of our primary interest, in which all the forces acting upon the particles balance each other in the microchannel. For this reason, rhombic and equilateral hexagonal cross-sections with 1 mm of the axial length were considered. Physical dimension of the channel cross-section was kept identical to those used in the experimental works for an appropriate comparison. Similar to the experiments, two particle diameters (i.e., *d* = 5 and 13 μm) and two representative flow rates (i.e., 1 and 10 μL/min) were examined in the current numerical study. It should be noted that numerical results for the rhombic microchannel is from our recent literature [24].

**Figure 2:**
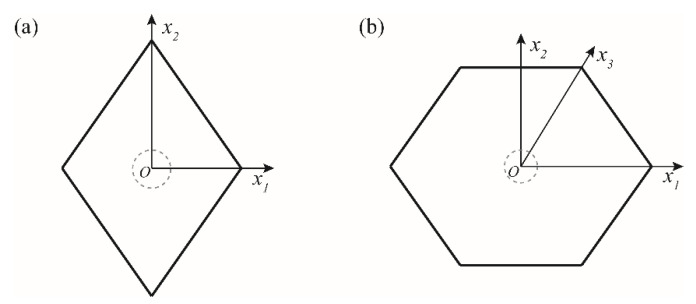
Cross-sectional shape of (**a**) the rhombic and (**b**) the equilateral hexagonal microchannel employed in the numerical simulation. $x_{1}$ and $x_{2}$ on a cross-plane denote a horizontal and a vertical axis in Cartesian coordinate system, respectively, while $x_{3}$ represents an axis from the origin to the corner bisector for an obtuse angle of the hexagonal microchannel.

Figure 2 shows cross-sectional shapes for both microchannels employed herein. Since those microchannels are non-circular, hydraulic diameter (*D_h_*) was chosen as a characteristic length scale. This allows us an appropriate comparison between two channels with respect to non-dimensional force balance although a side length and its resulting cross-sectional area for each channel are different (see Table 1). A uniform velocity and uniform pressure were applied for each inlet and outlet, which induces a centerline velocity of 0.069 m/s and 0.10 m/s for each rhombic and hexagonal microchannel at the flow rate of 10 μL/min. Therefore, the channel Reynolds number (*Re* = *ρ* *U_max_* *D_h_*_/_*μ*, where *ρ* is the fluid density, *U_max_* is the characteristic velocity, and *μ* is the mean viscosity, respectively) are 2.03 and 2.95 for each rhombic and hexagonal channel at the flow rate of 10 μL/min. The particle Reynolds number (Rep=Red/Dh2, where *d* is the particle diameter) is thus in a range of $O\left(10^{-3}\right)-O\left(10^{-1}\right)$. The flow and particle parameters in this numerical study are summarized in Table 2 below.

Although ANSYS Fluent provides reliable and robust computational approaches for various practical cases, there is no well-defined physical model for viscoelastic fluid. Instead, leveraging user-defined function (UDF) provided by the software package enables us to embed an approximated model for a particular viscoelastic fluid based upon the experimental data. Since the current working fluid used in the experiment is PEO500, which is considered as a viscoelastic fluid, it is important to model the viscosity depending on a range of shear rates in the present simulation. Lim et al., [31] performed viscosity measurements for the same PEO500 solution. Thus, a power-law model was obtained based upon the experimental result and successfully simulated the viscosity of PEO500 solution as was done in our previous work [24]. It should be noted that a lower boundary of the power-law model was set for $\dot{\gamma }>18$, although the experimental data shows a second decreasing tendency in viscosity followed by a certain plateau for $10<\dot{\gamma }<100$. Therefore, the lower boundary in this model was considered to be an average value between the viscosity corresponding to the plateau and the one at $\dot{\gamma }=1000$ in the experimental data so as to minimize the numerical uncertainty.

A single rigid spherical particle was set at the fixed location on the cross-sectional area for both rhombic and hexagonal microchannels (see Figure 2). A net lift force that acts on the particle was computed to explore an inertial focusing phenomenon in the present channels primarily along the line of symmetry. In particular, the two lines of symmetry were considered on a cross-plane of the rhombus such as *x*_1_ and *x*_2_, representing a horizontal and vertical axis in the Cartesian coordinate system, whereas the hexagonal case has an additional line of symmetry (*x*_3_) corresponding to the axis from the origin to the corner bisector for an obtuse angle of the hexagon. Thus, multiple locations along the line of symmetry provide a variation in a net lift force acting on the particle with respect to the channel center.

It is worth noting that the net lift force in this study is directly computed by ANSYS Fluent instead of calculating and combining individual force terms such as a wall-induced lift force, a shear-gradient lift force, and a viscoelastic force. The Magnus effect resulting from particle rotation and its corresponding pressure difference was not considered in the present net lift force since the spherical particle was fixed as an irrotational rigid body in the microchannel. Despite its significant contribution to the particle focusing, the Magnus effect can be often neglected when the particle Reynolds number (*Re_p_*) is less than 0.1 [32] that satisfies the current simulation regime. Therefore, analyzing the net lift force in this study allow us to estimate the underlying mechanism of the elasto-inertial particle focusing with respect to equilibrium position based on the magnitude and slope of the net lift force. Finally, the viscoelastic effect on the particle migration will be highlighted when comparing to the experimental and numerical results having the same channel geometry but different working fluid (Newtonian DI water) [33]. This will be discussed in further detail in the “Results and Discussion” section.

## 3. Results and Discussion

For an effective particle separation in a microchannel both in Newtonian and non-Newtonian regimes, the blockage ratio of the particle diameter and characteristic length of the channel (*β* = *d/D_h_*) plays a significant role, and it should be in a range of 0.1~0.2 based on the previous studies [20,34,35]. Two inertial particles, with a diameter of 5 and 13 μm, were considered in the present study in order to represent simplified red blood cells and white blood cells. Thus, we set the hydraulic diameter of the channel as a characteristic length to be 62 μm, with corresponding blockage ratio of 0.08 and 0.21 for each particle.

Under this notion in the experimental design, we fabricated microchannels with rhombic and equilateral hexagonal cross-sections to have the identical hydraulic diameter (*D_h_*) for an appropriate comparison. Table 2 shows their side lengths and calculated hydraulic diameters.

Particle focusing was observed in Newtonian and non-Newtonian fluid flows in microfluidic channels with rhombic and equilateral hexagonal cross-sections. Firstly, we investigated the particle focusing in Newtonian fluid and then observed the migration of particles in non-Newtonian viscoelastic fluid.

In Newtonian fluid, two dimensionless numbers are defined to describe the flow of particles [36,37]. One is the channel Reynolds number (*Re*) and the other is the particle Reynolds number (*Re_p_*). The Reynolds number (*Re*) is a dimensionless number, indicating the relative importance of the inertial effect and viscous effect (Equation (1)). The particle Reynolds number (*Re_p_*) is related to the fluid Reynolds number (*Re*) through the ratio of particle and channel length scales (Equation (2)), enabling to predict the flow velocity (*U_max_*) at which particles focus in microchannels. That is, flows with higher *Re_p_* can migrate the particles faster, therefore forming the focused particle streams at each equilibrium position.

In non-Newtonian viscoelastic fluid, however, particles are affected by additional elastic lift force due to the non-uniform normal stress differences [17]. The Weissenberg number (*Wi*) is a dimensionless number, characterizing the elastic effects of a non-Newtonian fluid in the channel (Equation (3)). For the particle focusing in non-Newtonian viscoelastic fluid, the Weissenberg number and Reynolds number are utilized to characterize the elastic effect and the inertial effect [15]. That is, the fluid elasticity (*El*), which is the relative importance of the elastic effect to the inertial effect, is defined as the ratio of Weissenberg number to Reynolds number (Equation (4)). *Re*, *Wi*, and *El* are calculated with the parameters on the middle plane in the flow direction. Through comparing these two dimensionless numbers (*Re* and *Wi*), it is possible to know a major force for the particle focusing phenomena.

*Re* = *ρ*
*U_max_*
*D_h/_μ* (1)

(2) $${Re}_{p}=Re{\left(d/D_{h}\right)}^{2}$$

(3) $$W_{i}\;=\;\lambda \dot{\gamma }\;=\;2\gamma V_{m}/w$$

*El* = *Wi/Re* (4)

where *ρ* is the fluid density, *U_max_* is the characteristic velocity, *D_h_* is the hydraulic diameter, *d* is the particle diameter, *μ* is the mean viscosity, *λ* is the relaxation time of polymer solution, $\dot{\gamma }$ is the shear rate, *w* is the microchannel width, *Q* is the flow rate.

### 3.1. Particle Focusing under Newtonian Fluids

**Figure 3:**
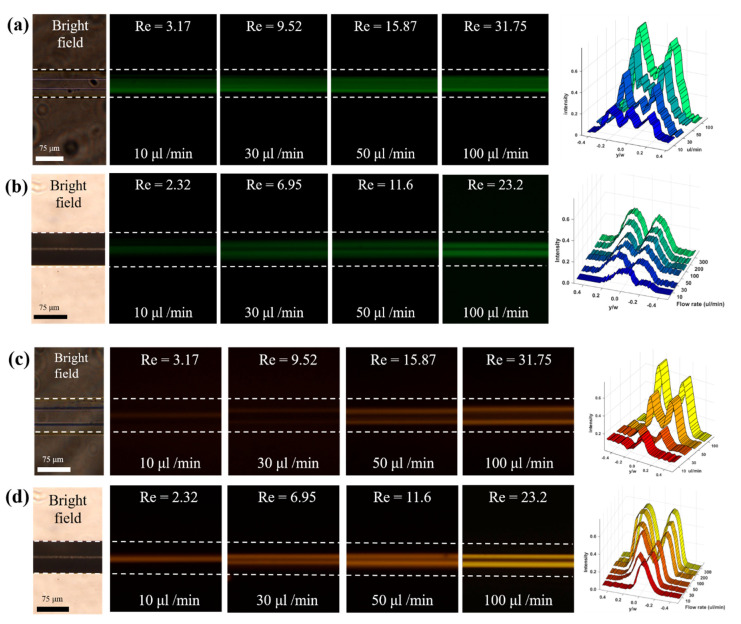
Fluorescence images and intensities graphs (top view) of particle focusing in non-rectangular microchannel under Newtonian fluid according to various flow rates. (**a**) equilateral hexagon (**b**) rhombus for 5 μm particle (green), and (**c**) equilateral hexagon (**d**) rhombus for 13 μm particle (red).

Figure 3 presents the focusing of particles (top view) in the microchannel with rhombic and equilateral hexagonal cross-sections for different flow rates and particle size under Newtonian fluid (DI water). The dotted lines illustrate the ends of the microchannel, which were obtained from the bright-field images under the same experimental conditions. For the rhombic microchannel, we could observe two focusing positions easily, but it was not easy to observe any focusing position for the equilateral hexagonal microchannel due to overlapped positions. It was confirmed that four particle focusing positions were measured using a confocal microscope. The particles were randomly distributed at low *Re_p_*, but the peaks representing the focusing position became clear with increasing *Re_p_*, as shown in Figure 3.

**Figure 4:**
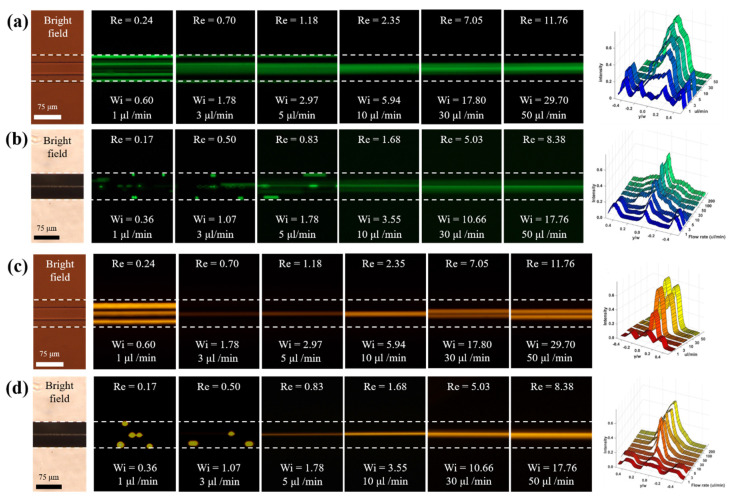
Fluorescence images and intensities graphs (top view) of particle focusing in non-rectangular microchannel under non-Newtonian according to various flow rates. (**a**) equilateral hexagon (**b**) rhombus for 5 μm particle (green), and (**c**) equilateral hexagon (**d**) rhombus for 13 μm particle (red).

Figure 3a,b shows that the particles with a 5 μm diameter are distributed and scattered around the center of the equilateral hexagonal and rhombic microchannel for low flow rates, less than 10 μL/min, because the inertial force is not strong enough to push entire particles toward their equilibrium positions. As the flow rates increase over 50 μL/min, two inertial focusing points were clearly observed in the rhombic microchannel due to the strong inertial force [30]. In the equilateral hexagonal microchannel, however, particles are still distributed and scattered around the center, showing the wider focusing bands. Although *D_h_* and *d* are the same for both microchannels, they have different *Re* for the same flow rate (*Q*) due to the different cross-section area, as shown in Table 2. Therefore, *Re_p_* of the equilateral hexagonal microchannel is smaller than that of the rhombic microchannel. However, the large particles with a 13 μm diameter show the two (rhombus) or four (equilateral hexagon) focusing points over 30 μL/min, as shown in Figure 4c,d. At high *Re_p_* for both microchannels, inertial force exerted on a particle was proportional to the fourth of a particle size so that tight and stable particle focusing could be achieved [4].

From the experimental results, it is concluded that particles in the rhombic microchannel could be more efficiently focused than that in the equilateral hexagonal microchannel at lower *Re*. It is also confirmed that the lateral migration and focusing of the particles under Newtonian fluid were mainly governed by inertial force which was affected by the particle size [4,20] rather than cross-sectional shape of the microchannel.

### 3.2. Particle Focusing under Non-Newtonian Fluids

Particle behavior under non-Newtonian viscoelastic fluid is totally different from that under Newtonian fluid. Figure 4 shows the fluorescence image of particle focusing in the non-rectangular microchannel under non-Newtonian fluid according to the flow rate and particle size. In elasto-inertial particle focusing, the blockage ratio is an important parameter so we set the hydraulic diameters of two non-rectangular microchannels to the same value during fabrication process. Therefore, it is easy to investigate and compare the effect of flow rate and blockage ratio on the elasto-inertial focusing for both microchannels.

For both microchannels, at low flow rates (*Re* ≈ 0 and *Wi* > 0), the equilibrium points of particles with a 5 μm diameter (*β* = 0.08) were located at the channel center and corners up to the flow rate of 3 μL/min (Figure 4a,b). Therefore, the particles moved either along the center or the corners of the equilateral microchannel (5 peaks) and rhombic microchannel (3 peaks) owing to the negligible inertial force and relevant elastic force. As the flow rate increased up to 10 μL/min (*Re* > 0 and *Wi* > 0), the particles were focused at the center of both microchannels under the influence of elasto-inertial force, but the particles were not completely focused in the center, showing a band shape. This is due to the result of competition between the non-negligible inertial lift force and relevant elastic force under viscoelastic fluid flowing through straight microchannel with non-rectangular cross-sectional shape [20,21]. The microchannel with rhombic cross-section shows tighter particle focusing than that with the equilateral hexagonal cross-section.

For particles with relatively large particle size (13 µm diameter), as shown in Figure 4c,d, the effect of elastic-inertia force was dominant from 3 µL/min, and the particles were well focused in the center of the equilateral hexagonal microchannel. However, the particles were well focused in the center of the rhombic microchannel from 5 µL/min. When the flow rate increased and reached 30 µL/min (inertial force dominant regime), single-line particle focusing changed to double-line focusing in the equilateral hexagonal microchannel but there was no change in the rhombic microchannel even though for higher flow rates. That is, in the rhombic microchannel, the focused particles just dispersed a little around the centerline but kept single-line focusing at high flow rates (up to 100 μL/min). It will be discussed in detail together with simulation results in Section 3.3.

Consequently, particle migration and focusing were more distinct at the high blockage ratio (*β* = 0.21) because the force on the particle induced by the normal stress is proportional to the cube of the particle diameter [20]. Different particle focusing positions were observed for both microchannels according to the flow rates. Specifically, in the equilateral hexagonal microchannel, the number of focusing positions decreased from five to one to two by transitioning from a pure elastic to an elasto-inertial to inertial regime. For both microchannels, single-line particles focusing in viscoelastic fluids can be simply realized through the control of flow rate without any external force, which could not be achieved under Newtonian fluid.

### 3.3. Simulation Results

**Figure 5:**
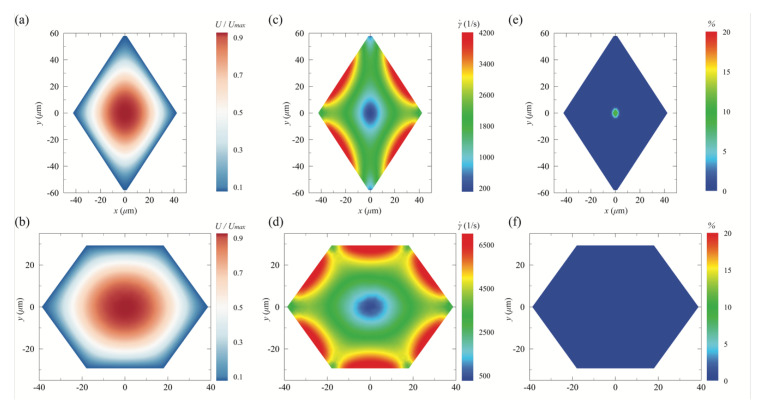
Simulation results of the (**a**,**b**) streamwise velocity magnitude (m/s), (**c**,**d**) shear rate (1/s), and (**e**,**f**) percent discrepancy in the shear rate between the current viscoelastic fluid (PEO500 solution) and DI water in a cross section of the rhombic and equilateral hexagonal microchannel at 10 µL/min.

Figure 5a–d presents the magnitude of the streamwise velocity and shear rate resulting from the numerical simulation with viscoelastic fluid for both rhombic and hexagonal microchannels at the flow rate of 10 µL/min. In averaging sense, both channels show similar trend in the velocity and shear rate on their cross-planes. For example, the local maximum of the streamwise velocity occurs at the channel center and gradually decreases toward the wall as expected (see Figure 5a–d). The contour maps of the shear rate for both microchannels are shown in Figure 5c,d. The shear rate as a differential of the fluid velocity is of particular importance in elasto-inertial particle focusing since it is highly associated with the competing mechanism of the inertial lift force between shear gradient and wall interaction [36]. In the viscoelastic regime, furthermore, inertial particles tend to migrate toward the region where the shear rate is zero, particularly when the second normal stress difference in a viscoelastic fluid is negligible [36,38]. As seen in Figure 5c,d, it is clear that both channel centers have a zero-shear rate region and the shear rate becomes near zero at each corner for both cases. Based on the previous experiment [31], PEO500 solution is well-known viscoelastic fluid that yields a shear-thinning characteristic. However, it has a negligible second normal stress difference so that a secondary flow on a channel cross-section is not induced. Therefore, particle focusing that occurs in the current rhombic and hexagonal microchannels with viscoelastic fluid (PEO500) is primarily attributed to the combined effect between the fluid inertia (related with the shear rate) and the elasticity in a certain fluid condition (see Figure 4).

To further evaluate a contribution factor in particle migration, percent discrepancy in shear rate was made between the present viscoelastic fluid and DI water at the same flow rate (i.e., 10 µL/min) as follows:

$$\Delta \dot{\gamma }\left(\%\right)=\frac{\left|{\dot{\gamma }}_{visc}-{\dot{\gamma }}_{water}\right|}{{\dot{\gamma }}_{visc}}\times 100,$$

where ${\dot{\gamma }}_{visc}$ and ${\dot{\gamma }}_{water}$ denote the shear rate for the present non-Newtonian working fluid and DI water, respectively. This discrepancy provides a difference in shear rate between those two fluid cases. As shown in Figure 5e,f, it is clear that the shear rate is almost the same between the current working fluid and DI water for both microchannels. This means that the viscoelastic effect on elasto-inertial particle focusing can be isolated when comparing with a particle migration pattern of DI water at the same channel geometry at the same flow rate. For example, inertial particles were better focused at both channel centers in the viscoelastic regime at the flow rate of 10 µL/min (see Figure 4) as compared to the results with Newtonian DI water (see Figure 3). Based on our result in the percent discrepancy, it can be said that the difference in migration pattern between Figure 3 and Figure 4 is due mainly to the presence of viscoelastic effect in different working fluids.

**Figure 6:**
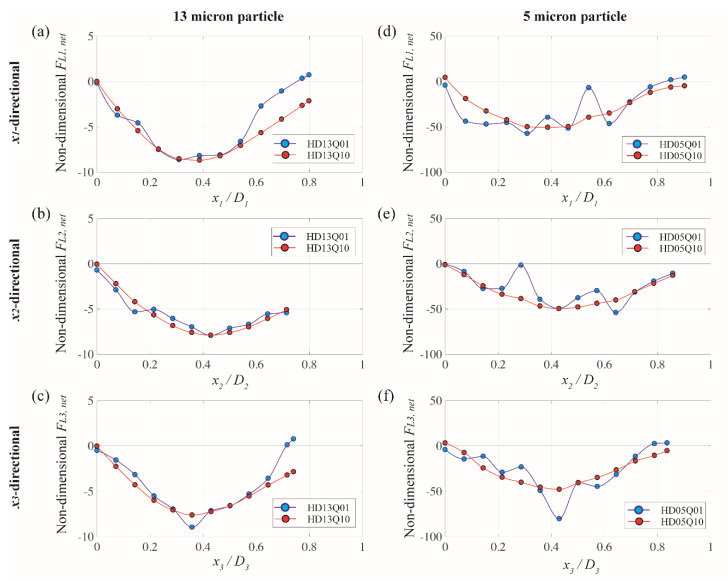
Variation of (**a**,**d**) *x*_1_-directional, (**b**,**e**) *x*_2_-directional, and (**c**,**f**) *x*_3_-directional net elasto-inertial force exerted on 5 and 13 µm particles along the line of symmetry for the equilateral hexagonal microchannel. Blue and red symbols represent the case for the flow rate of 1 and 10 µL/min, respectively.

Figure 6 displays a variation of *x*_1_-, *x*_2_-, and *x*_3_-directional net elasto-inertial force (F*_L,net_*) for 5 and 13 µm particles along the lines of symmetry for the equilateral hexagonal microchannel, as was referred in Figure 2b. The net elasto-inertial force was first normalized with $\rho_{f}U_{max}^{2}d^{4}/D_{h}$, where $\rho_{f}$ and *U_max_* denote the fluid density and the centerline velocity of the channel. Horizontal axis in the figure was normalized by the distance from the channel center to the wall for each *x_i_*-direction. Blue and red symbols represent the case for the flow rate of 1 and 10 µL/min, respectively. Solid lines were made by piecewise cubic interpolation in order to show a shape-preserving trend in F*_L,net_* along the lines of symmetry for all cases. It should be also noted that F*_L,net_*, after a certain point, was not resolved simply because the spherical particle started to touch the channel wall.

**Figure 7:**
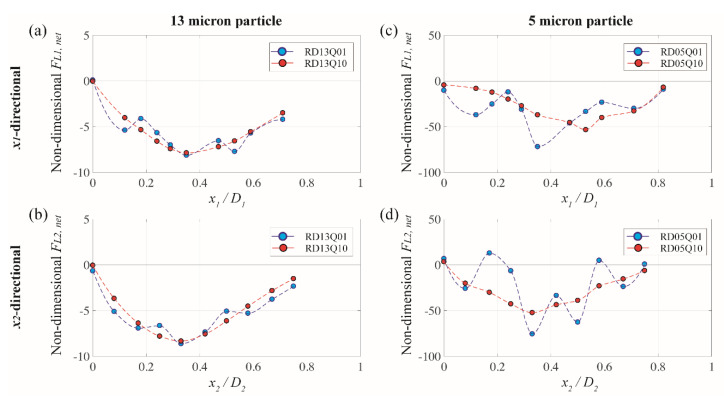
Variation of (**a**,**b**) *x*_1_-directional, and (**c**,**d**) *x*_2_-directionall net elasto-inertial force exerted on 5 and 13 µm particles along the line of symmetry for rhombic microchannel. Blue and red symbols represent the case for the flow rate of 1 and 10 µL/min, respectively.

In Figure 6, negative value in F*_L,net_* indicates a force direction toward the channel center, while positive value represents a directional force to the corner bisector for *x*_1_- and *x*_3_-directions and to the wall for *x*_2_-direction, respectively (see Figure 2). For the case of 13 µm particle and the flow rate of 10 µL/min (HD13Q10, red symbols in Figure 6a–c), the net elasto-inertial forces are mainly negative and have a single zero point at the channel center for all directions. This means that 13 µm particles suspended in viscoelastic fluid are likely to migrate toward the channel center independent of their initial position. Such behavior results from a combined effect of inertia and elasticity when the moderate flow condition is maintained [20,39]. This migration pattern at the flow rate of 10 µL/min in viscoelastic regime is consistently observed in the numerical result of rhombic channel (RD13Q10), as shown in Figure 7a,b) [24], and shows a good agreement with the experimental result in Figure 4c,d).

When the fluid inertia becomes smaller (i.e., 1 µL/min of the flow rate herein), particle migration pattern on a channel cross-plane significantly changes. By comparing between blue (*low* fluid inertia) and red (*high* fluid inertia) symbols in Figure 6a–c, several blue symbols are located in positive F*_L,net_* at the end of the line for the case of *x*_1_- and *x*_3_-directions where the corner bisector exists. This produces a zero-crossing from negative to positive value in F*_L,net_* and corresponds to an additional equilibrium point close to the corner, in which the shear rate approaches near zero. This numerical observation agrees well with the previous study that when the fluid inertia becomes negligibly small, particle migration occurs to the channel center and the closest corner depending on their initial positions and Weissenberg number [39]. This observation also explains a unique migrating behavior of 13 µm particles at the flow rate of 1 µL/min, particularly for the hexagonal microchannels in the experiment (see Figure 4c).

For the case of rhombic microchannel with a smaller fluid inertia (RD13Q01) shown in Figure 7a,b, a variation of net forces shows a similar trend as compared to those of the hexagonal cases. This means that 13 µm particles are also supposed to move toward a centerline of the rhombus where c ~ 0. However, the experimental result at the same flow rate (see Figure 4d) does not show a single train of fluorescent particles at the channel center. Instead, particles seem to be stuck inside the channel. As was discussed in our previous study [24], this is because of the lack of fluid inertia (i.e., *U_max_* = 6.9 × 10^−3^ m/s) that does not sufficiently drift particles toward the lateral equilibrium point. To be more specific, the present numerical simulation computes the net lift force based on a fixed spherical particle. Thus, the net lift forces herein only indicate us a direction of the force but does not care of whether inertial particles actually migrate or not based on a given fluid inertia.

Figure 6d–f presents net elasto-inertial forces along the line of symmetry in *x*_1_-, *x*_2_-, and *x*_3_-direction for the particles with relatively smaller size (5 µm diameter). Although such plots become more fluctuating when the flow rate decreases, the overall trend in plots with respect to the equilibrium point is quite consistent to those with large particles (13 µm diameter) in Figure 6a,b. For example, another equilibrium point close to the corner bisector exists in addition to the channel center at the flow rate of 1 µL/min, owing possibly to a strong elastic effect rather than inertial force, whereas a higher flow rate induces comparable effect between inertial and elastic forces that provide a single equilibrium point at the channel center. The experimental result in Figure 4a confirms this observation, as the fluorescent image of 5 µm particles at the flow rate of 1 µL/min shows five peaks in intensity and becomes a single-peak when the flow rate increases to 10 µL/min.

For the small-sized particles particularly at the flow rate of 10 µL/min, it is interesting to note that the pattern of *x*_1_-directional net force in the hexagonal channel (HD05Q10, see Figure 6d) is different from the one for the rhombic one (RD05Q10, see Figure 7c). For the case of the rhombic channel, F*_L1,net_* (red symbol) stays near zero at the channel center and gradually increases as is the particle position moves away from the center. Also, the lateral position of a local minimum is shifted toward the corner (i.e., *x*_1_/D_1_ ~ 0.55) as compared to the one of HD05Q10 (i.e., *x*_1_/D_1_ ~ 0.55). Therefore, particles in this rhombic channel case still migrate toward the channel center as a single equilibrium position occurs at the center. However, particles may be less strongly clustered at the equilibrium position owing to a relatively smaller magnitude in F*_L1,net_* and a shifted local minimum in comparison with the one of HD05Q10. Experimental observation in Figure 4b supports this numerical finding since the fluorescence image of 5 µm particles at the flow rate of 10 µL/min shows a wider focusing band than the hexagonal case at the same particle and the same flow rate. Thus, the influence of the elastic-inertia force on particle migration is more effective at the obtuse corner that is embodied in the hexagonal channel compared to the acute corner of the rhombic one.

**Figure 8:**
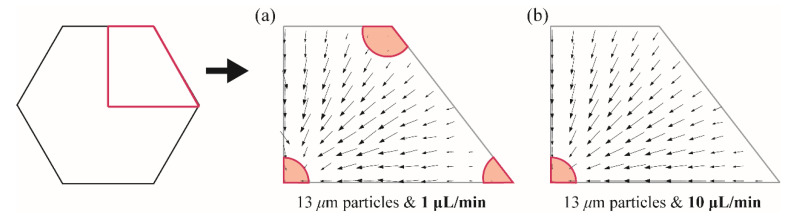
Vectors of the net elasto-inertial force in equilateral hexagonal microchannel for 13 µm particles at (**a**) the flow rate of 1 µL/min and (**b**) the flow rate of 10 µL/min.

Finally, Figure 8 displays vectors of the net elasto-inertial force in equilateral hexagonal microchannel for 13 µm particles at two different flow rates. Each vector denotes (F*_L1,net_*, F*_L2,net_*) at each point that varies with the particle position inside the channel. Figure 8 qualitatively shows an equilibrium point on a channel cross-section depending on the fluid inertia (i.e., flow rate). As seen in Figure 8a, the case of HD13Q01 clearly shows three equilibrium points at the channel center and each corner. When increasing the flow rate (HD13Q10), particles may consistently migrate to the channel center independent of their initial position on a cross-section as all vectors are directed toward the center as seen in Figure 8b.

## 4. Conclusions

In this work, two kinds of non-rectangular PDMS microchannels with rhombic and equilateral hexagonal cross-sections were fabricated using basic MEMS processes, hot-embossing, micromolding and self-alignment of PDMS molds. We carried out the experiments for the inertial and elasto-inertial particle focusing behaviors under Newtonian fluid and non-Newtonian fluids according to the flow rate and particle size. The experimental results showed that the single-line particle focusing could be formed in the centerline of the microchannel in the non-Newtonian fluid. That is, under the combined effects of inertia and elasticity, elasto-inertial particle focusing were realized at the center of the rhombic and equilateral hexagonal microchannel without any external force. In addition, we numerically studied the elasto-inertial focusing to investigate the effects of cross-sectional geometry accompanied with viscoelasticity on the focusing phenomenon. Simulation results for particle migration induced by viscoelastic effect agrees well with experimental results in both qualitative and quantitative ways. By comparing the results between two types of microchannel, it was confirmed that the influence of elastic-inertia force on particle focusing is more effective for the microchannel that embodies the obtuse corner rather than the one embodying the acute corner, although the characteristic length scale was kept identical for both channels. It is expected that this PDMS microfluidic device with non-rectangular microchannel can be used as a high-throughput and cost-effective microfluidic device for particle/cell separation and sorting.

## Figures and Tables

**Table 1:** Side length, calculated hydraulic diameter and cross-sectional area of microchannels with rhombic and equilateral hexagonal cross-sections.

	Side Length	Hydraulic Diameter (*D_h_*)	Cross-Sectional Area
Rhombic channel	80.7 μm	62 μm	5000 μm^2^
Equilateral hexagonal channel	36 μm	62 μm	3340 μm^2^

**Table 2:** Flow and particle parameters in the numerical simulation.

Case	Channel Cross-Section	*d* (μm)	Flow Rate (μL/min)	*U_max_* (m/s)	Re	Re*_p_*
RD05Q01	Rhombus	5	1	6.9 × 10^−3^	0.20	1.3 × 10^−3^
RD05Q10	Rhombus	5	10	6.9 × 10^−2^	2.03	1.3 × 10^−2^
RD13Q01	Rhombus	13	1	6.9 × 10^−3^	0.20	8.9 × 10^−3^
RD13Q10	Rhombus	13	10	6.9 × 10^−2^	2.03	8.9 × 10^−2^
HD05Q01	Hexagon	5	1	1.0 × 10^−2^	0.29	1.9 × 10^−3^
HD05Q10	Hexagon	5	10	1.0 × 10^−1^	2.95	1.9 × 10^−2^
HD13Q01	Hexagon	13	1	1.0 × 10^−2^	0.29	1.3 × 10^−2^
HD13Q10	Hexagon	13	10	1.0 × 10^−1^	2.95	1.3 × 10^−1^

## Data Availability

This study does not report any data.
